# QUALITATIVE COMPARISON OF TWO INTERGENERATIONAL SERVICE-LEARNING PROGRAMS

**DOI:** 10.1093/geroni/igac059.1529

**Published:** 2022-12-20

**Authors:** Sara Bartlett, Sky Bergman, Phyllis Solomon, Zvi Gellis

**Affiliations:** Cal Poly, San Luis Obispo, San Luis Obispo, California, United States; Cal Poly, San Luis Obispo, San Luis Obispo, California, United States; University of Pennsylvania, Philadelphia, Pennsylvania, United States; University of Pennsylvania, Philadelphia, Pennsylvania, United States

## Abstract

Intergenerational service-learning programs are an effective and frequently used training modality in undergraduate education and are often examined using qualitative methods. It is less common to qualitatively examine two such programs to compare their outcomes. This study reports qualitative findings from a mixed-methods study comparing two intergenerational service-learning programs in an undergraduate Psychology of Aging class. The longer, more relational intervention, the Lives Well Lived program, matched students and older adults exemplifying “successful aging” in a mutual interviewing, life review project utilizing documentary film, photography, and memoir creation. The comparison intervention, the Meals That Connect/Lunch Bunch program, also exposed students to older adults exemplifying successful aging, but in a shorter, less relational way. A convenience sample of 128 students (65 in the intervention group and 63 in the comparison group) answered post-intervention open-ended questions about what they liked/disliked about the program in which they participated, as well as any viewpoints about aging they felt changed or were reinforced by the project. Thematic analysis revealed students in both groups experienced decreased ageism and improved attitudes about aging. However, those participating in the Lives Well Lived program had closer relationships with the older adults participating in the project, expressed more positivity about their own aging process, and indicated more willingness to engage in future intergenerational relationships. Use of a comparison project in qualitative examination of intergenerational service-learning adds greater insight into such programs’ outcomes, enhancing quantitative effectiveness examinations.

